# Cationic antimicrobial peptides serve as activation signals for the *Salmonella* Typhimurium PhoPQ and PmrAB regulons *in vitro* and *in vivo*

**DOI:** 10.3389/fcimb.2012.00102

**Published:** 2012-07-27

**Authors:** Susan M. Richards, Kristi L. Strandberg, Megan Conroy, John S. Gunn

**Affiliations:** Department of Microbial Infection and Immunity, Center for Microbial Interface Biology, The Ohio State University, ColumbusOH, USA

**Keywords:** *Salmonella* Typhimurium, CAMPs, PhoPQ, PmrAB, lipopolysaccharide modification

## Abstract

*Salmonella enterica* serovar Typhimurium uses two-component regulatory systems (TCRSs) to respond to environmental stimuli. Upon infection, the TCRSs PhoP-PhoQ (PhoPQ) and PmrA-PmrB (PmrAB) are activated by environmental signals detected in the lumen of the intestine and within host cells. TCRS-mediated gene expression leads to upregulation of genes involved in lipopolysaccharide (LPS) modification and cationic antimicrobial peptide (CAMP) resistance. This research expands on previous studies which have shown that CAMPs can activate *Salmonella* TCRSs *in vitro*. The focus of this work was to determine if CAMPs can act as environmental signals for PhoPQ- and PmrAB-mediated gene expression *in vitro*, during infection of macrophages and in a mouse model of infection. Monitoring of PhoPQ and PmrAB activation using recombinase-based *in vivo* expression technology (RIVET), alkaline phosphtase and β-galactosidase reporter fusion constructs demonstrated that *S*. Typhimurium PhoQ can sense CAMPs *in vitro*. In mouse macrophages, the cathelecidin CRAMP does not activate the PhoPQ regulon. Acidification of the *Salmonella*-containing vacuole activates PhoP- and PmrA-regulated loci but blocking acidification still does not reveal a role for CRAMP in TCRS activation in mouse macrophages. However, assays performed in susceptible wild type (WT), CRAMP knockout (KO), and matrilysin (a metalloproteinase necessary for activating murine α-defensins) KO mice suggest CRAMP, but not α-defensins, serve as a putative direct TCRS activation signal in the mouse intestine. These studies provide a better understanding of the *in vivo* environments that result in activation of these virulence-associated TCRSs.

## Introduction

Upon infection, bacteria must evade the arsenal of host immune defenses, including cationic antimicrobial peptides (CAMPs), designed to eliminate invading pathogens. CAMPS are structurally diverse innate immune molecules providing protection against infection for all classes of life (Menendez and Brett Finlay, 2007). CAMPS are amphipathic peptides that are classified based on their secondary structure, and can be separated into categories including α- or β-defensins and cathelicidins. The antimicrobial activity of CAMPs comes from the ability of these molecules to insert into the microbial membrane, resulting in membrane destabilization and microbial lysis (Matsuzaki et al., 1997; Radek and Gallo, 2007; Bucki et al., 2010). These peptides are rapidly produced by macrophages and epithelial cells in response to infection or injury and can mediate inflammation and stimulate the immune system upon detection of pathogens (Menendez and Brett Finlay, 2007).

The Gram-negative enteric pathogen *Salmonella enterica* serovar Typhimurium (*S*. Typhimurium) can use two-component regulatory systems (TCRSs), such as PhoP-PhoQ (PhoPQ) and PmrA-PmrB (PmrAB), to detect environmental signals and to mediate changes in gene expression that promote survival and virulence (Miller et al., 1989; Roland et al., 1993; Gunn and Miller, 1996). Indirect activation of PmrAB occurs through the PhoPQ TCRS. PhoPQ activates the expression of PmrD, which can regulate PmrA activity at the post-transcriptional level by binding to and stabilizing PmrA in its phosphorylated form (Kox et al., 2000; Kato and Groisman, 2004). In addition to known *in vitro* signals such as low magnesium (Mg^2+^), acidic pH and high concentrations of iron (Fe^3+^), *S.* Typhimurium PhoPQ and PmrAB and their regulons are also activated by unknown environmental signals in macrophages, in other host cells and in the intestinal lumen (Foster and Hall, 1990; Alpuche Aranda et al., 1992; Garcia Vescovi et al., 1996; Bearson et al., 1998; Wosten et al., 2000; Merighi et al., 2005).

CAMPs also have been shown to activate PhoPQ *in vitro* and may be *in vivo* factors involved in *Salmonella* TCRS-mediated gene expression and LPS modification (Bader et al., 2003). Several CAMPs bind to acidic patches on the inner membrane-facing region of the PhoQ periplasmic domain, resulting in PhoQ conformational changes and activation of the PhoPQ regulon (Bader et al., 2005). Therefore, *Salmonella* may sense and respond to CAMPs in the environment and during infection through PhoQ to prevent killing by these host molecules and other immune defenses (Bader et al., 2003, 2005). PmrAB-regulated modification of LPS lipid A with positively-charged molecules such as aminoarabinose (Ara4N) and phosphoethanolamine promotes CAMP resistance by reducing the anionic charge of the bacterial outer membrane (Gunn et al., 2000; Tamayo et al., 2005). PhoPQ-regulated lipid A modification also promotes resistance to CAMP killing.

Merighi et al. used recombinase-based *in vivo* expression technology (RIVET) analysis to examine *in vivo* expression of PhoP- and PmrA-regulated genes (Merighi et al., 2005). These authors found that the PhoPQ-regulated gene *pagP* [palmitoyl transferase that mediates palmitate addition to *Salmonella* lipid A (Belden and Miller, 1994; Guo et al., 1998)] and the PmrAB-regulated gene *pmrH* [first gene in the seven gene operon involved in Ara4N addition to the lipid A (Gunn et al., 1998b, 2000)] are expressed early during *Salmonella* infection in response to unknown factors in the *in vivo* environment. Known *in vitro* activating signals, acidic pH and high iron concentrations, were not responsible for TCRS-mediated gene activation (Merighi et al., 2005). These authors also showed that TCRS-mediated gene activation *in vivo* requires the presence of active PmrA and PhoP, as *pmrA* and *phoP* mutants did not express *pmrH* in response to the *in vivo* environment (Merighi et al., 2005).

The complex interplay between host immune factors and bacterial defense systems during the early stages of *Salmonella* infection is still poorly understood. CAMPS are likely to be one of the earliest-encountered components of the immune system. They protect the host against infection both directly, through potent bactericidal activity, and indirectly, by inducing chemotaxis of monocytes and neutrophils to the site of infection. Bader et al. proposed a model in which CAMP detection by *Salmonella* leads to TCRS-induced signaling that could result in regulation of virulence genes, as well as increased resistance to these innate immune molecules and other host defenses through LPS modification (Bader et al., 2003, 2005). We further hypothesize that *S*. Typhimurium is able to detect and respond to sublethal levels of CAMPS through the PhoPQ/PmrAB TCRS *in vivo*, in the lumen of the intestine and within host cells.

## Materials and methods

### Bacterial strains and growth conditions

*S.* Typhimurium strains used in this study are listed in Table 1. The *pmrI*::*Mud*J mutant strains listed in Table 1 were originally generated by random *Mud*J mutagenesis in a *pmrA*^*c*^ background (as previously described by Tamayo et al.) and then were transduced into WT and PhoP^−^ strains of *S.* Typhimurium to create the *pmrI*::*Mud*J and *pmrI*::*Mud*J PhoP^−^ strains used in this study (Tamayo et al., 2002). Luria-Bertani (LB) broth and agar were used for strain maintenance and experimentation. When appropriate, antibiotics were added at the following concentrations: ampicillin, 50 μg/ml; chloramphenicol, 25 μg/ml; kanamycin, 50 μg/ml; tetracycline, 15 μg/ml. Two of the CAMPs tested were murine CRAMP and the human cathelicidin ortholog, LL-37 (Gudmundsson et al., 1996; Gallo et al., 1997). Other molecules of interest were the cationic lipopeptide polymyxin B (PMB), which targets Gram-negative bacteria in a manner similar to CAMPs, and a PMB derivative, polymyxin B nonapeptide (PMBN) (Vaara and Vaara, 1983). PMBN is used to examine gene activation by cationic molecules, but it cannot penetrate bacterial membranes or cause cell death due to the lack of a fatty acid tail (Vaara and Vaara, 1983). A range of peptide concentrations was used initially for all *in vitro* assays and concentrations of 0.5–5 μg/ml of PMBN, CRAMP and LL-37 showed similar results. Results from 5 μg/ml (5 μM PMBN, 1.3 μM CRAMP, and 1.1 μM LL-37) were reported in most of the assays as this represented the highest, yet non-lethal concentration in this range. For PMB, a range of 0.01–0.1 μg/ml was tested and all showed similar results. Results from 0.06 μg/ml (0.04 μM) were reported in the assays in this work.

**Table 1:** ***Salmonella enterica* serovar Typhimurium strains**.

**Strain**	**Relevant characteristics**	**Source or reference**
JSG162	CS019; *pagJ*::Tn*phoA* (Kan/Cam)	Belden and Miller, 1994
JSG163	14028s; *pagJ*::Tn*phoA phoP*105::Tn10d (Kan/Tet)	Belden and Miller, 1994
JSG174	CS019; *pagP*::Tn*phoA* (Kan/Cam)	Belden and Miller, 1994
JSG2860	*phoN*::Tn*phoA* (Kan)	Miller et al., 1989
JSG1051	*pmrI*::*Mud*J	This study
JSG1070	*pmrI::Mud*J PhoP^−^	This study
JSG1071	*pmrC*::*Mud*J	Miller et al., 1989
JSG2428	*pmrH*::*tnpR* RIVET strain; JSG246 + pJSG2413 cointegrated at pmrH=> Phi(pmrH′-tnpRmut135-lacZ+)566 res1-tet-res1 = akaMM566 (Amp/Tet)	Merighi et al., 2005
JSG2502	*pagP*::*tnpR*RIVET strain; JSG246 + pJSG2483; f(pagP′-tnpRmut135-lacZ+)608-6 res1-tet-res1 (Amp/Tet)	Merighi et al., 2005
JSG2579	*fepA*::*tnpR*RIVET strain; JSG246 + pJSG2535; Phi(fepA′-tnpRmut135-lacZ+)2535 res1-tet-res1 (Amp/Tet)	Merighi et al., 2005

### Alkaline phosphatase (AP) detection of PhoPQ activation

*S*. Typhimurium AP reporter strains capable of displaying AP activity when Tn*phoA*-containing gene products are translated [*pagJ*::Tn*phoA* (JSG162), *pagJ*::Tn*phoA* PhoP^−^ (JSG163), *pagP*::Tn*phoA* (JSG174), and *phoN*::Tn*phoA* (JSG2860)] were used to measure PhoPQ two-component system activation following CAMP exposure. *S.* Typhimurium strains were grown in a rotating drum at 37°C with aeration to early log phase (all cultures were normalized to an optical density at 600 nm [OD_600_] of 0.2), and incubated with 1.1 μM LL-37 (AnaSpec, Fremont, CA), 1.3 μM CRAMP (Anaspec), 0.04 μM PMB (Sigma-Aldrich, St. Louis, MO), or 5 μM PMBN (Sigma-Aldrich) for 90 min at 37°C with aeration to induce PhoPQ-mediated gene expression. These methods were based on the protocol described by Bader et al. (2005). After incubation with the CAMPs, a standard AP assay protocol was followed. Miller units, representing relative AP activity, were calculated according to the following equation: Miller units = (1000 × OD_420_)/(OD_600_ × Sample volume (0.1 mL) × Time lapsed until color change).

### β–Galactosidase detection of PmrAB activity

CAMP-induced activation of PmrAB-regulated gene expression was quantified using *S.* Typhimurium Mu*d*J reporter fusion strains (JSG1051, JSG1070 and JSG1071). *S.* Typhimurium strains were grown as for the AP assays and incubated with 1.1 μM LL-37, 1.3 μM CRAMP, 0.04 μM PMB or 5 μM PMBN for 90 min at 37°C with aeration to induce PmrAB-mediated gene expression. β-galactosidase assays were carried out using a spectrophotometric method with *ortho*-nitrophenyl-β-galactoside (ONPG) as a substrate. Miller units, representing relative β-galactosidase activity, were calculated as for the AP assays.

### RIVET *in vitro* assays

The *S.* Typhimurium RIVET strains (*pagP* [JSG2502], *pmrH* [JSG2428] or *fepA* [control, JSG2579]) were used to measure PhoPQ and PmrAB-mediated gene activation by the heritable loss of bacterial Tet resistance. The RIVET strains were grown as described for the AP assays and then were incubated for 4 h or 24 h in the presence or absence of sublethal concentrations of LL-37 (1.1 μM), CRAMP (1.3 μM) or PMB (0.04 μM), as well as low (10 μM) or high (10 mM) Mg^2+^, and enumeration plating was employed to determine bacterial survival. Resolution of antibiotic resistance cassettes from RIVET strains was quantified through patching individual colonies (*n* ≤ 100) onto LB or LB_tet_ and calculating percent sensitivity.

### RIVET assay in murine macrophages

All tissue culture experiments were incubated at 37°C plus 5% CO_2_. Bone marrow-derived macrophages (BMDMs) were obtained from the femurs of WT (BALB/c) and background-matched CRAMP knockout (KO) mice and were cultured in Iscove's Modified Dulbecco's Medium [(IMDM), Gibco, Grand Island, NY)] containing 10% heat-inactivated fetal bovine serum (FBS), 50% L929-conditioned IMDM, 0.6% non-essential amino acids (Gibco), 100 U/ml penicillin, and 100 mg/ml streptomycin (Gibco)] at 37°C in a humidified incubator containing 5% CO_2_ for 5 days to allow for monocytes to differentiate into macrophages (Swanson and Isberg, 1993; Stanley, 1997; Amer and Swanson, 2005; Abdelaziz et al., 2011). After 5 days, BMDMs were counted and 2 × 10^5^ cells were seeded into each well of a 24-well tissue culture plates (BD Falcon, Franklin Lakes, NJ), and incubated overnight to allow for BMDM adherence. BMDMs were then infected with *S.* Typhimurium grown in a rotating drum at 37°C with aeration to late log phase (cultures were adjusted to an OD_600_ of 0.9) at an MOI of 10:1 (bacteria: macrophage). For experiments with bafilomycin, bafilomycin A1 from *Streptomyces griseus* (Sigma-Aldrich) was added to macrophage wells at a final concentration of 100 nM. For experiments with ammonium chloride [NH_4_Cl (Sigma-Aldrich)], it was resuspended in IMDM and added to macrophage wells at a final concentration of 10 mM. Tissue culture wells were centrifuged briefly following the addition of bacteria to synchronize infection. BMDMs were incubated with bacteria at 37°C for 2 h to allow adequate time for phagocytosis of *S.* Typhimurium, and media was then removed from each well and replaced with IMDM plus 10% FBS containing either 50 μg/ml or 10 μg/ml gentamicin (Gibco) to kill extracellular *S.* Typhimurium. Wells that received 50 μg/ml gentamicin represent the initial infection timepoint and were incubated for an additional 30 min to allow for killing of extracellular bacteria, after which the BMDMs were processed for enumeration of intracellular *S.* Typhimurium. Wells that received 10 μg/ml of gentamicin were processed for enumeration of intracellular *S.* Typhimurium after 6, 10 and 24 h of *S.* Typhimurium exposure. BMDMs were lysed by 0.01% Triton-X-100. Intracellular survival was determined by enumeration of macrophage lysates on LB agar. Gene activation of RIVET strains was calculated as a percentage of colonies having resolved the Tet cassette as described previously. The percentage of activation in medium alone was subtracted from the percentage of Tet-sensitive colonies recovered from macrophage lysates at each time point.

### Mouse assays

Experiments with WT and CRAMP KO mice were performed in 6–10 week old female BALB/c (WT) mice from Harlan Sprague Dawley (Indianapolis, IN), as well as background-matched CRAMP KO mice obtained from Dr. Bradford McGwire at The Ohio State University. Female C57BL/6 mice and B6.129-Mmp7 (MMP7 KO) mice (6–10 weeks old) were purchased from The Jackson Laboratories (Bar Harbor, ME). Mice were used in accordance with guidelines established by The Ohio State University Institutional Animal Care and Use Committee (IACUC). Food and water were removed from the mouse cages 4 h prior to infection. Mice were orally infected (in triplicate) with 1 × 10^8^ colony forming units (CFU) *S.* Typhimurium in 100 μl PBS. After infection, food and water were returned to the cages. Mice were euthanized at 4, 12, 24 and 48 h post-infection (p.i.) For experiments in BALB/c and CRAMP KO mice, the intestine lumen contents, 2–4 Peyer's patches near the distal ileum and the spleen were removed from each mouse and homogenized in 1 mL of cold PBS. For experiments in C57BL/6 and MMP7 KO mice, 2–3 inches of the small intestine closest to the caecum (distal ileum) and the spleen were removed from each mouse, homogenized in PBS diluted, plated, incubated and patched as above (see RIVET Assay in Murine Macrophages). The percentage of Tet-sensitive colonies obtained from each sample/condition was calculated to determine the level of *in vivo* promoter activation.

### Statistics

For all experiments, each condition was analyzed in duplicate or triplicate in 2–5 independent experiments. The results obtained were averaged and then analyzed for statistical significance using the two-tailed Student's *t*-test.

## Results

### Cationic antimicrobial peptides differentially activate the *Salmonella* PhoPQ and PmrAB regulons *in vitro*

**Figure 1:**
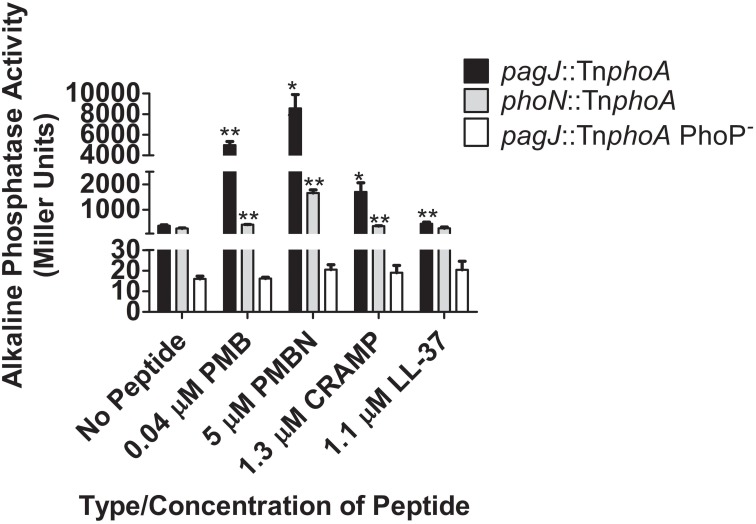
**PhoPQ-mediated activation of *S*. Typhimurium *phoN* and *pagJ* in response to CAMPs**. AP assays demonstrate that different CAMPs induce varying levels of PhoP-regulated gene promoter expression. CAMP sensing by PhoQ and subsequent PhoP-mediated AP reporter gene activation is abolished in the absence of PhoP. Values shown represent the mean ± standard deviation from a representative experiment performed at least twice in triplicate. Statistical significance was measured against the samples to which no peptide was added. ^*^Indicates *p* < 0.05, ^**^Indicates *p* < 0.005.

Previous research has showed that certain CAMPs can activate the expression of PhoP-regulated genes (Bader et al., 2003, 2005; Shprung et al., 2012). To further examine *Salmonella* PhoPQ-mediated CAMP activation *in vitro*, we performed AP assays with *S*. Typhimurium strains containing a *phoA* (AP) reporter fusion to PhoP-regulated genes *phoN* and *pagJ* (Kier et al., 1979; Miller et al., 1989; Belden and Miller, 1994; Gunn et al., 1998a). The *S*. Typhimurium AP reporter strains were grown in the presence or absence of PMB, PMBN, CRAMP or LL-37 for 90 min. A significant increase in PhoPQ-regulated promoter expression was observed when *S.* Typhimurium was grown in the presence of all four peptides (*phoN* increased with LL-37 but not significantly), compared to growth in the absence of each CAMP (Figure 1). PMB and PMBN induced the highest levels of *pagJ* and *phoN* promoter-driven AP activity among the CAMPs examined. Interestingly, *pagJ* and *phoN* show different levels of induction in response to the various CAMPs used. To verify that the observed *in vitro* CAMP sensing by *Salmonella* was dependent on PhoP-mediated gene activation, AP assays were also performed with a *pagJ*::Tn*phoA* PhoP^−^ reporter fusion strain (Bader et al., 2003), where AP activity in the presence of peptides was greatly reduced in comparison to the WT *pagJ* reporter strain (Figure 1). These experiments confirm previous work published by Bader et al. demonstrating that specific CAMPs play a role in the expression of the PhoPQ regulon *in vitro*. In addition, PhoP is required for gene activation in the presence of CAMPs (Bader et al., 2003, 2005).

**Figure 2:**
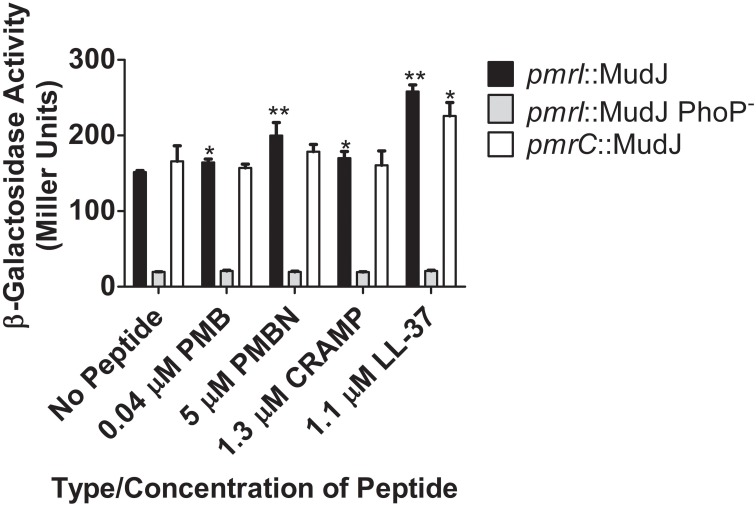
**TCRS-mediated activation of *S*. Typhimurium *pmrI* and *pmrC* in response to CAMPs**. β-galactosidase assays demonstrate that different CAMPs induce varying levels of PmrA-regulated gene promoter expression and CAMP sensing by PhoQ. Subsequent PmrA-mediated β-galactosidase reporter gene activation is abolished in the absence of PhoP. Values shown represent the mean ± standard deviation from a representative experiment performed at least twice in triplicate. Statistical significance was measured against the samples to which no peptide was added. ^*^Indicates *p* < 0.05; ^**^indicates *p* < 0.005.

To measure the ability of CAMPs to activate genes in the PmrAB regulon, β-galactosidase assays were performed with the *S*. Typhimurium strains containing the *pmrI* or *pmrC* promoter fused to the *lacZ* reporter gene by the Mu*d*J transposon (JSG1051, JSG1070 and JSG1071). PMB, PMBN, CRAMP and LL-37 all were able to induce statistically higher levels of β-galactosidase activity from the *pmrI* promoter compared to *S.* Typhimurium grown without peptide (Figure 2). The observed *pmrI*-regulated reporter gene activation in the presence of CAMPs was dramatically reduced in the absence of PhoP (Figure 2). The *pmrC*::Mu*d*J reporter strain also showed increased β-galactosidase activity in the presence of PMBN and LL-37 but not the other CAMPs tested (Figure 2). These findings indicate that, as observed with the PhoPQ-regulated genes in the AP assays, PmrAB-regulated genes can be induced in response to CAMPs, but this induction is not uniform or observed with all CAMPs to which *Salmonella* was exposed.

**Figure 3:**
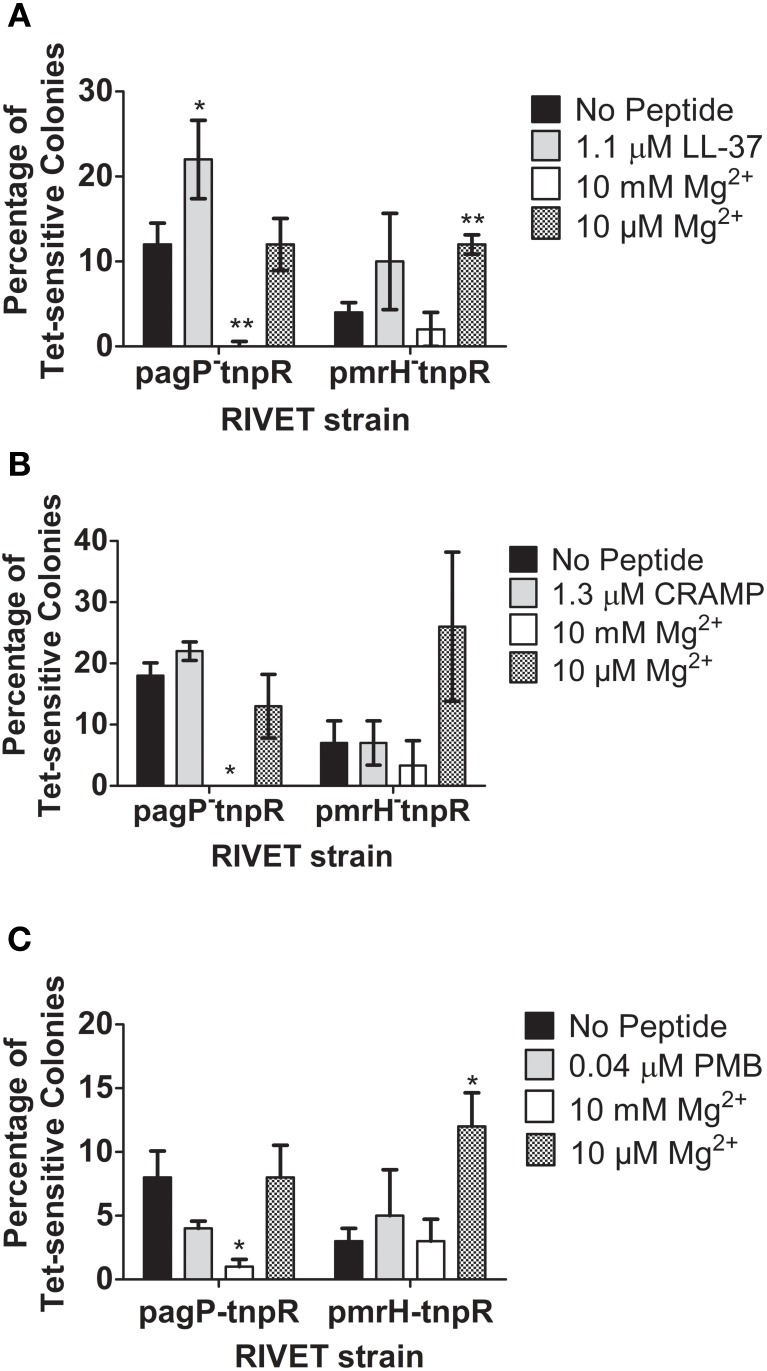
**Induction of *pagP* and *pmrH* in response to *S*. Typhimurium TCRS-mediated sensing of LL-37 and magnesium**. RIVET strains containing the promoter regions of a PhoP-mediated gene, *pagP*, or a PmrA-regulated gene, *pmrH*, were incubated *in vitro* with **(A)** LL-37, **(B)** CRAMP or **(C)** Polymyxin B and high (10 mM) or low (10 μM) MgCl_2_ (Mg^2+^) for 4 h to induce resolvase production. Bacteria were diluted and plated on LB O/N at 37°C and patched on LB and LB + Tet for detection of Tet-sensitive colonies. The percentage of recovered Tet-sensitive colonies from each sample was averaged for analysis. Graphed values represent the mean ± standard deviation of one representative experiment performed at least twice in triplicate. ^*^Indicates *p* < 0.05; ^**^indicates *p* < 0.005.

To further confirm that PhoQ can induce TCRS-mediated gene activation in response to CAMPs *in vitro*, the *S*. Typhimurium *pagP* and *pmrH* RIVET strains were incu-bated for 4 h or 24 h (24 h data not shown due to similar trends) in the presence or absence of sublethal concentrations of LL-37, CRAMP and PMB (Gallo et al., 1997). Addition of high (10 mM) or low (10 μM) Mg^2+^ served as a control for appropriate regulation of PhoPQ and PmrAB by a known signal. Exposure to high Mg^2+^ resulted in the absence of Tet-resistance colonies suggesting that there is complete repression of the regulons under these environmental conditions. Results from *in vitro* experiments performed using the *fepA* control RIVET strain did not show changes in the heritable loss of bacterial Tet resistance (data not shown). In general, the RIVET strains demonstrated little PhoPQ or PmrAB activation in the presence of sublethal concentrations of CAMPs *in vitro*. The *pagP* promoter was activated to a higher level than was the *pmrH* promoter; however, this greater activation was only seen with LL-37 under the conditions tested. LL-37 (1.1 μM) induced the highest level of *S*. Typhimurium TCRS-mediated signaling compared to the no peptide control, while CRAMP (1.3 μM) and PMB (0.04 μM) did not activate *pagP* or *pmrH* significantly in these experiments (Figures 3A–C). The observed greater *pagP* activation supports the idea that PhoQ is the direct sensor of CAMPs, since PhoQ activation of direct target genes would be stronger than downstream activation of *pmrH* that occurs through PhoP modulation of PmrA (Gunn et al., 1998b; Kato et al., 2003).

Taken together, the AP, β-galactosidase assays and RIVET assays confirm that CAMPs can induce expression of PhoP- and PmrA-regulated genes *in vitro*. Also, different CAMPs were shown to have variable abilities to activate the PhoPQ and PmrAB regulons in the *in vitro* assays performed. Differences in positive charge, hydrophobicity and amphipathicity may account for the varying abilities of the tested CAMPS to activate PhoPQ and PmrAB (Shprung et al., 2012).

### CRAMP is not a significant macrophage intracellular signal for PhoPQ or PmrAB activation

**Figure 4:**
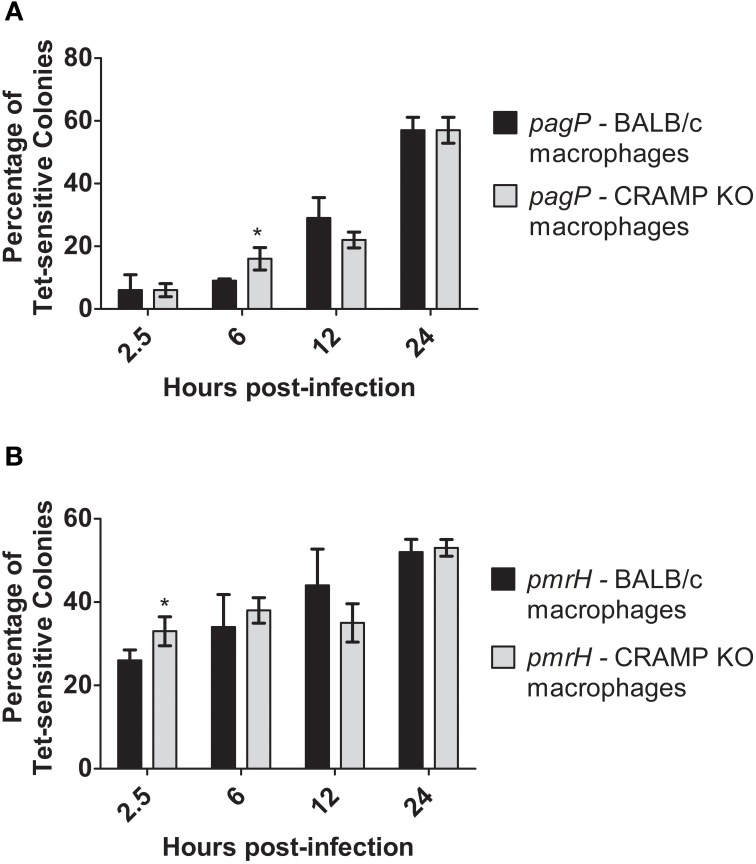
**TCRS-mediated activation of *pagP* and *pmrH* in WT and CRAMP KO macrophages**. BALB/c and CRAMP-deficient mouse bone marrow-derived macrophages were infected with the *S.* Typhimurium **(A)** *pagP* or **(B)** *pmrH* RIVET strain. Gentamycin-treated macrophages were lysed at 2.5, 6, 12 and 24 h p.i., Lysates containing intracellular bacteria were plated on LB and incubated O/N at 37°C. Resulting colonies were patched onto LB and LB Tet to detect loss of Tet-resistance due to promoter activation. Graphed values represent the mean ± standard deviation of one representative experiment performed at least twice in triplicate. ^*^Indicates *p* < 0.05.

CRAMP has been shown to be expressed in the intestines and has also been found to be produced by murine macrophages in response to *Salmonella* infection *in vitro* (Gallo et al., 1997; Rosenberger et al., 2004). Despite generally low levels of CAMP-mediated RIVET reporter gene activation *in vitro*, macrophages were infected with the RIVET strains to determine if CAMPs can induce *pagP* or *pmrH* expression in host environments that *Salmonella* is known to encounter during infection. To examine intracellular PhoP- and PmrA-regulated gene activation, BMDMs from WT (BALB/c) and background-matched CRAMP KO mice were infected with the *S*. Typhimurium RIVET strains at an MOI of 10:1 (bacteria: macrophage). As a control, patching of *S*. Typhimurium RIVET strains after overnight growth of the initial cultures used for infection confirmed that these strains are not activated in LB or IMDM. As expected, resolution dramatically increased for *pagP* and *pmrH* over the 24 h macrophage infection period (Figures 4A,B). Early time points (≤6 h p.i.) surprisingly showed slightly higher resolution in macrophages from CRAMP KO vs. WT macrophages, which was contrary to our hypothesis concerning the involvement of CRAMP in PhoPQ-mediated signaling. At later time points p.i., there were no significant differences in the percentage of Tet-sensitive colonies recovered. In addition, there were no differences in recovery of total bacteria at these time points from CRAMP KO vs. WT macrophages that would account for biases in the results (data not shown). Thus, CRAMP alone does not appear to be responsible for the observed activation of the PhoPQ or PmrAB TCRSs in mouse macrophages. In addition, the equal recovery of bacteria from CRAMP KO vs. WT macrophages demonstrate that CRAMP does not kill *S*. Typhimurium during macrophage infection.

### pH reduction activates *S*. Typhimurium TCRSs inside murine macrophages

Acidic pH is an activating signal for the PhoPQ or PmrAB TCRSs *in vitro* and should be encountered by *S.* Typhimurium while inside the macrophage phagosome (Alpuche Aranda et al., 1992). To examine the role of pH as a signal for intramacrophage TCRS activation, as well as its potential synergy with CRAMP, bafilomycin A1 (Baf) or ammonium chloride (NH_4_Cl) was added to the medium to inhibit the acidification p.i., in the murine macrophage phagosome (Gordon et al., 1980; Lukacs et al., 1990; Tapper and Sundler, 1995).

**Figure 5:**
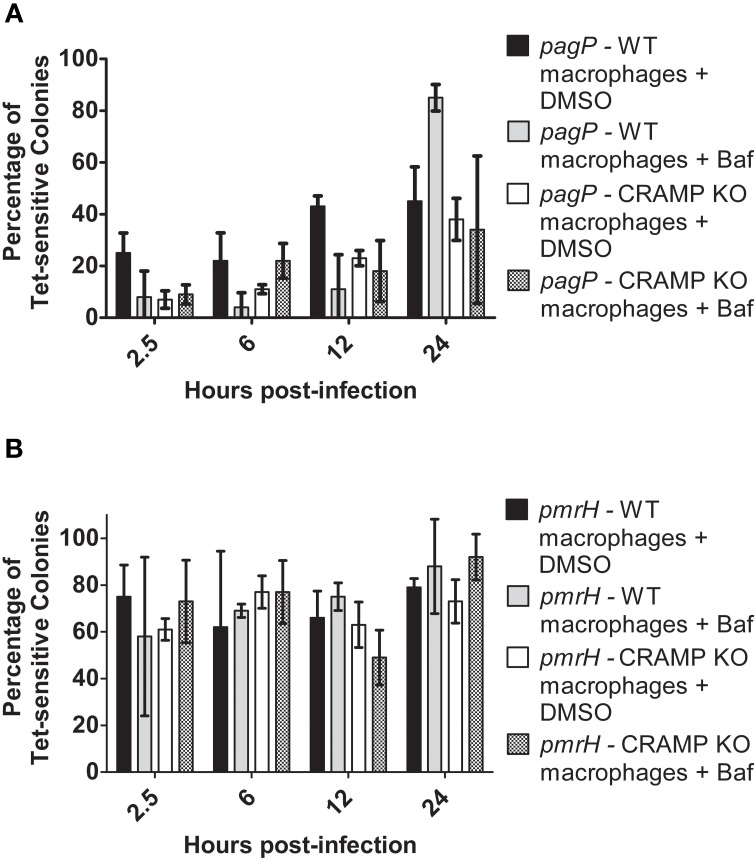
**TCRS-mediated *pagP* and *pmrH* activation in bafilomycin-treated WT and CRAMP KO macrophages**. BALB/c and CRAMP-deficient mouse bone marrow-derived macrophages were pre-treated with bafilomycin in DMSO and infected with the *S.* Typhimurium **(A)** *pagP* or **(B)** *pmrH* RIVET strain. Gentamicin-treated macrophages were lysed at 2.5, 6, 12 and 24 h p.i., Lysates containing intracellular bacteria were plated on LB. Resulting colonies were patched onto LB and LB Tet to detect loss of Tet-resistance due to promoter activation. Graphed values represent the mean ± standard deviation of one representative experiment performed at least twice in triplicate.

**Figure 6:**
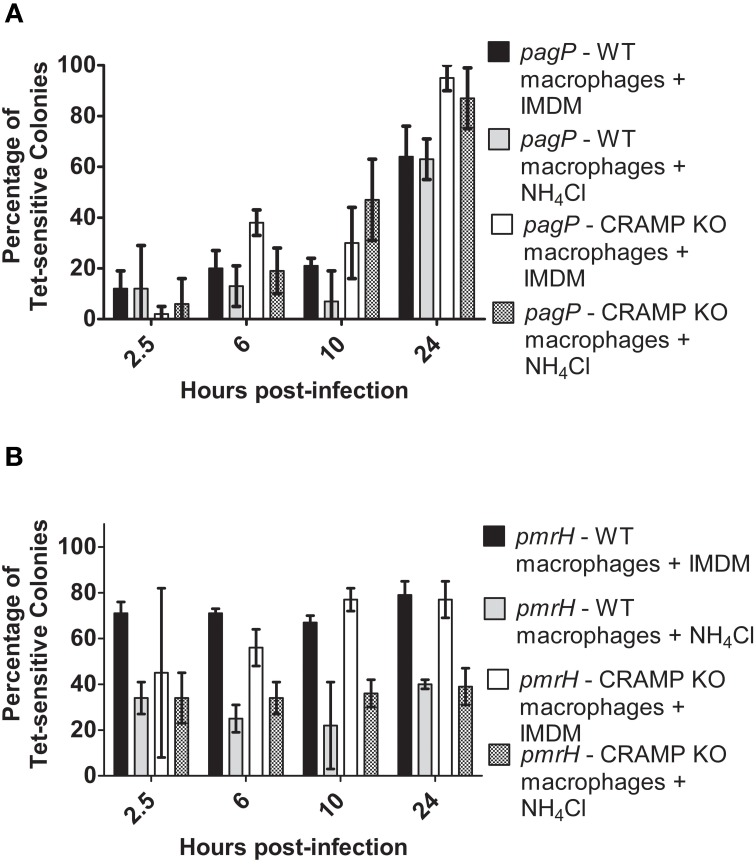
**TCRS-mediated *pagP* and *pmrH* activation in NH_4_Cl-treated WT and CRAMP KO macrophages**. BALB/c and CRAMP-deficient mouse bone marrow-derived macrophages were pre-treated with NH_4_Cl in IMDM and infected with the *S.* Typhimurium **(A)** *pagP* or **(B)** *pmrH* RIVET strain. Gentamicin-treated macrophages were lysed at 2.5, 6, 10 and 24 h p.i. Lysates containing intracellular bacteria were plated on LB. Resulting colonies were patched onto LB and LB Tet to detect loss of Tet-resistance due to promoter activation. Graphed values represent the mean ± standard deviation of one representative experiment performed three times in triplicate.

Again, resolution increased for *pagP* and *pmrH* over the 24 h infection period as expected, (Figures 5A,B) but not as dramatically as seen in the experiments in Figure 4. At all time points except 24 h p.i., the addition of Baf resulted in lower recovery of Tet-sensitive colonies in the *pagP* RIVET fusion strain (i.e., reduced PhoPQ activation; Figure 5A). The addition of Baf to CRAMP KO macrophages showed no additional decrease in fusion activity, consistant with the data in Figure 4 suggesting the lack of a role for CRAMP in intramacrophage PhoPQ activation. Regarding *pmrH* fusion activity, there was no significant difference in the percentage of Tet-sensitive colonies recovered under any of the conditions examined (Figure 5B). The use of NH_4_Cl instead of Baf to abrogate phagosome acidification showed similar trends to the results with Baf (Figure 6A), with the exception that NH_4_Cl treatment dramatically decreased fusion activity for *pmrH* (Figure 6B). Thus, pH plays a role in the induction of *pagP* and *pmrH* gene expression over the course of infection in mouse macrophages, but CRAMP does not appear to play a role in PhoP or PmrA-mediated gene expression even in the absence of phagosome acidification.

### *S*. Typhimurium can sense murine CRAMP within the mouse intestine

Previous work from our lab has demonstrated that *S.* Typhimurium isolated from the lumen of the distal ileum displayed increased levels of *pmrH* and *pagP* expression (Merighi et al., 2005). To expand upon these findings, CAMP-deficient mouse models were used to determine if antimicrobial peptides could serve as environmental factors promoting *pagP* and *pmrH* expression in the small intestine. To examine this, BALB/c (WT) and CRAMP KO as well as C57BL/6 (WT) and MMP7 KO (matrilysin-deficient) mice were infected with *S*. Typhimurium *pagP* and *pmrH* RIVET strains. Matrilysin is a cysteine protease found in intestinal crypts that cleaves the pro-form of cryptdins into their active form (Wilson et al., 1999).

**Figure 7:**
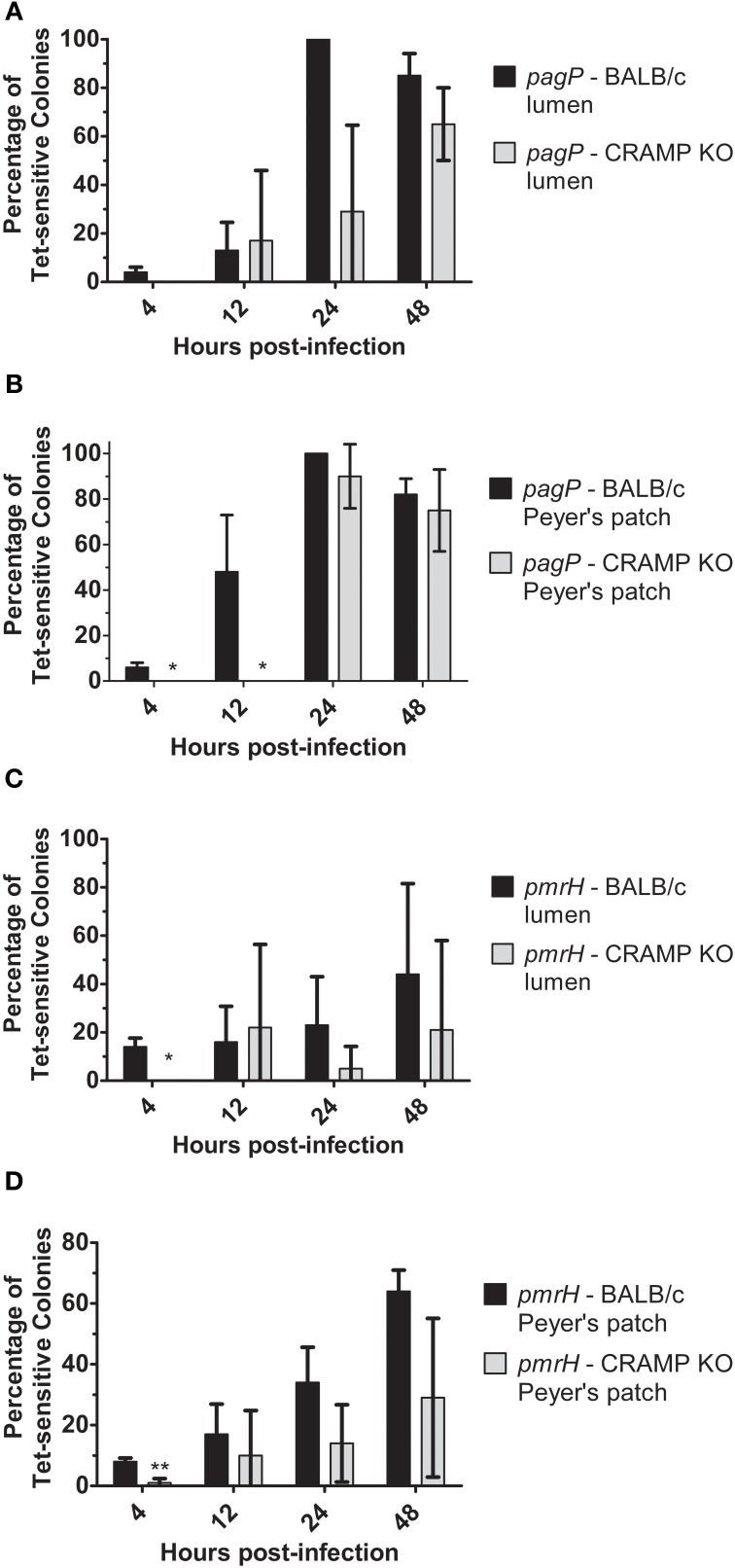
**TCRS-mediated activation of *S*. Typhimurium *pagP* and *pmrH* in the WT and CRAMP KO murine intestinal lumen and Peyer's patches**. BALB/c and CRAMP KO mice were infected orally with 10^8^ CFU of the *S.* Typhimurium *pagP* or *pmrH* RIVET strains. Mice were sacrificed at 4, 12, 24 and 48 h p.i. for removal of infected organs. Tissue samples were homogenized, diluted, plated on LB and grown overnight at 37°C to recover intracellular bacteria. One hundred colonies recovered from each sample were patched on LB and LB Tet to to detect loss of Tet-resistance due to promoter activation *in vivo*. Graphed values represent the mean percentage ± standard deviation of Tet-sensitive colonies recovered from the **(A)** and **(C)** intestinal lumen and **(B)** and **(D**) Peyer's patches of three BALB/c and three CRAMP KO mice infected with the *pagP* or *pmrH* RIVET strain for each time point in one representative experiment performed at least twice in triplicate. One asterisk (^*^) indicates *p* < 0.005 and two asterisks (^**^) indicate *p* < 0.001.

Examination of *in vivo* TCRS-mediated gene expression was performed at early and late time points after infection and focused on PhoPQ and PmrAB activation in the intestinal environment (lumen/Peyer's patches). The results in WT and CRAMP KO mice indicate that CRAMP plays a role in TCRS-mediated gene activation *in vivo*. Expression of *pagP* generally increased over time in both WT and CRAMP KO mice but was activated more frequently in WT compared to CRAMP KO mice in both the intestinal lumen and Peyer's patches at most time points (Figures 7A,B). The general trend for *pmrH* was that its activation also occurred in Peyer's patches and the intestinal lumen in both WT and CRAMP KO mice, but was activated more frequently in WT compared to CRAMP KO mice in both locations at most time points (Figures 7C,D). Thus, while CRAMP did not appear to play a dramatic role in activation of the PhoPQ/PmrAB TCRS in macrophages *in vitro*, may promote TCRS activation in intestinal/Peyer's patch tissues during infection.

**Figure 8:**
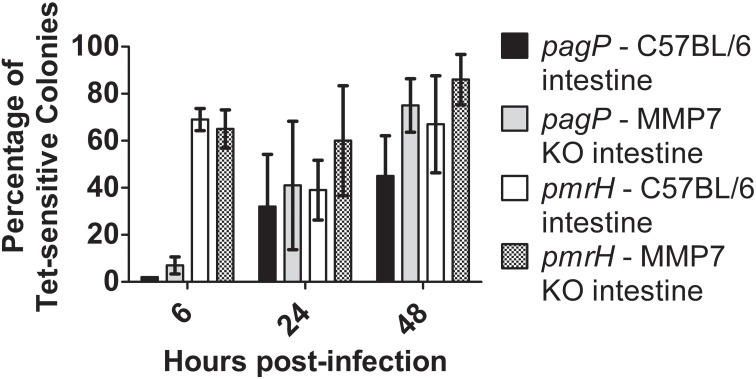
**TCRS-mediated activation of *S*. Typhimurium *pagP* and *pmrH* in the WT and Matrilysin-deficient murine intestine**. C57BL/6 and MMP7 KO mice were infected orally with 10^8^ CFU of the *S.* Typhimurium *pagP* and *pmrH* RIVET strains. Mice were sacrificed at 6, 24 and 48 h p.i., for removal of two-three inches of the small intestine (measured from the distal ileum). Tissue samples were homogenized, diluted, plated on LB and grown overnight at 37°C to recover intracellular bacteria. One hundred colonies recovered from each sample were patched on LB and LB Tet to detect loss of Tet-resistance due to promoter activation *in vivo*. Graphed values represent the mean percentage ± standard deviation of Tet-sensitive colonies recovered from the intestine of three BALB/c and three CRAMP KO mice infected with the *pagP* or *pmrH* RIVET strain for each time point in one representative experiment.

As observed with the RIVET experiments performed in WT and CRAMP KO mice (Figure 7), the *S*. Typhimurium *pagP* promoter exhibited increased activation in both C57BL/6 and background-matched MMP7 KO (matrilysin-deficient) mice over time (Figure 8). The *pmrH* promoter again showed early activation (as observed in previous tissue/macrophage experiments in this work) that was sustained at later time points. However, these data indicate that *Salmonella* does not directly respond to active cryptdins in the intestine, as there is no significant difference in RIVET reporter resolution in these mice.

## Discussion

The role of CAMPs as environmental signals for the expression of *Salmonella* PhoPQ- and PmrAB-regulated genes was examined using reporter fusions *in vitro*, in macrophages and in mice. Taken together, the results confirm that sublethal concentrations of CAMPs can activate *Salmonella* TCRS-mediated gene expression, as reported by Bader et al. (2003, 2005). This activation most likely occurs through the binding of CAMPs to the periplasmic domain of the inner membrane-bound protein kinase PhoQ, resulting in a signal transduction cascade that promotes expression of the PhoPQ and downstream PmrAB regulons (Bader et al., 2003, 2005).

Specific CAMPs were found to play a role in the activation of the PhoPQ and PmrAB regulons *in vitro*. The AP and β-galactosidase assays showed a significant increase in reporter activity when *S*. Typhimurium was grown in the presence of PMB, PMBN, CRAMP and LL-37, compared to growth in the absence of these CAMPs. The tested CAMPs induced different levels of PhoP-regulated gene activation, and CAMP-mediated signaling through PhoQ was shown to be dependent on the presence of PhoP. These findings suggest that PhoQ may respond differently to specific CAMPs and/or that PhoP may mediate the differential expression of genes in the PhoPQ regulon. These possibilities may result from variations in peptide binding to PhoQ or in promoter binding and activation by PhoP, as has been described by Kato et al. (Kato and Groisman, 2008). Variations in CAMP characteristics, such as net positive charge, can also effect TCRS activation, and may play a role in the differential activation of PhoPQ seen in these studies (Shprung et al., 2012).

Until recently, the paradigm regarding expression of the *Salmonella* PhoPQ and PmrAB regulons *in vivo* was that these TCRSs specifically detect intracellular environmental signals present in acidified phagosomes and therefore were not activated until bacteria enter into host macrophages (Alpuche Aranda et al., 1992). However, Merighi et al. performed RIVET experiments demonstrating that *S*. Typhimurium PhoQ and PmrB also recognize and respond to unknown extracellular host factors, based on expression of PhoP- and PmrA-regulated genes by *Salmonella* recovered from the small intestinal lumen of mice (Merighi et al., 2005) RIVET, which measures TCRS-mediated gene activation by the heritable loss of bacterial Tet-resistance, was used in this work to expand on the findings of Merighi et al. and determine if PhoQ or PmrB can detect CAMPs in the murine intestine and inside murine macrophages (Merighi et al., 2005).

Using the RIVET reporter *in vitro*, the results presented in this work indicate that LL-37, but not CRAMP and PMB, significantly activates PhoPQ- or PmrAB-regulated virulence genes under the tested experimental conditions. This suggests that the RIVET system may be less sensitive than the other reporter constructs used in this work (AP and β-galactosidase). Despite high sensitivity seen with the *in vitro* assays, AP and β-galactosidase fusions are less well suited to *in vivo* experimentation for which the RIVET technique was specifically designed. While the artificial conditions created in the laboratory may closely mimic local environments during infection, *in vitro* assays cannot account for synergistic effects of multiple CAMPs and additional environmental signals *in vivo* that may be detected that further induce the RIVET fusions. For this reason, *in vivo* and *ex vivo* experiments were conducted with RIVET strains, despite a possible reduced sensitivity of detection.

RIVET fusions to *pagP* and *pmrH* were clearly induced over time in macrophage cell culture as has been previously demonstrated (Merighi et al., 2005). Interestingly, *pmrH* was consistently highy activated early (within 2.5 h of infection), while *pagP* expression rose incrementally over time. This finding was also observed in human monocyte-derived macrophages (data not shown). This observation might suggest the sensing of non-induced phagolysosomal cues by PmrB while PhoQ senses signals that accumulate over time.

CRAMP, the murine cathelecidin and human LL-37 ortholog, has previously been implicated in *Salmonella* killing in mouse macrophages, as a PhoP-null strain survived better in CRAMP KO macrophages than WT macrophages (Rosenberger et al., 2004). This data suggests that CRAMP interacts with *Salmonella* within macrophages. However, CRAMP was not found in our studies to be significantly or consistently involved in activation of PhoQ- or PmrB-regulated genes in macrophages. Furthermore, we saw no differences in strain survival in CRAMP KO macrophages vs. WT macrophages (data not shown). As acidification of the *Salmonella* containing vacuole occurs post-phagocytosis, inhibitors of acidification (bafilomycin and NH_4_Cl) were used to determine if low pH, a known *in vitro* activating signal of both PhoQ and PmrB, had an effect on gene activation (Perez and Groisman, 2007; Prost and Miller, 2008). The results show a general effect of low pH on activation of *pagP* and *pmrH* fusions in macrophages, with bafilomycin treament having the greatest effect on *pagP* while NH_4_Cl had the greatest effect on *pmrH*. However, consistant with our previous data in this work, CRAMP did not play a role even in the absence of acidification of the vacuole.

While CRAMP is primarily localized in macrophages, it has also been reported to be found in the intestine and intestinal lumen (Gallo et al., 1997). Thus, we examined RIVET fusion induction in Peyer's patch tissue and in the intestinal lumen after oral infection of mice. These data demonstrated a general effect of CRAMP in both locations, though not always reaching statistical significance or occurring at every time point examined. The variability seen here and with other experiments is often inherent in experiments relying on mouse infections. Thus, CRAMP appears to activate *Salmonella pmrH* and *pagP* loci in the intestine, likely prior to entrance into macrophages, and may be the luminal/intestinal signal observed in RIVET experiments reported by Merighi et al. (2005). This is further supported by the fact that induction *in vivo* was dependent upon PhoPQ, which is the reported sensory system of CRAMP. It should be noted that the observed gene activation could result from the direct interaction of CRAMP with *S*. Typhimurium TCRSs and/or induction of other environmental factors or immune responses that are regulated by CRAMP. A complex indirect effect *in vivo* may explain why CRAMP demonstrated no role in PhoPQ/PmrAB activation *in vitro* in cultured macrophages.

We also examined active intestinal α-defensins (cryptdins) as a potential luminal signal of *pagP* and *pmrH* induction. MMP7 KO mice were used in these studies, as this protease is required to cleave the mouse α-defensins from a pro-form to an active form. Similar induction of *pagP* and *pmrH* was observed in the intestines of both WT and MMP7 KO mice, suggesting a non-role for the cryptdins in TCRS induction. While it is possible that non-processed cryptdins could be released from Paneth cells and participate in PhoPQ induction, it is unlikely based on our current understanding of the secretion of these CAMPs (Wilson et al., 1999).

Taken together, the results of this project and related findings by other researchers highlight the intricacy, and often redundancy, of *S*. Typhimurium TCRS-mediated gene activation and LPS modification in response to environmental sensing. Tight control of these bacterial defense mechanisms allows *Salmonella* to respond quickly and efficiently to constant changes in local stressors and host immune factors, including CAMPs. The findings presented here also highlight the benefit of performing intramacrophage and *in vivo* experiments, despite weak results *in vitro*. Media conditions in a test tube cannot represent accurately the complex interplay between host and pathogen. Further investigation into bacterial virulence gene regulation, surface modification and evasion of host defenses will help to determine how pathogens evade killing and thrive in the various hostile environments encountered during infection.

### Conflict of interest statement

The authors declare that the research was conducted in the absence of any commercial or financial relationships that could be construed as a potential conflict of interest.
